# Genome Stability Pathways in Head and Neck Cancers

**DOI:** 10.1155/2013/464720

**Published:** 2013-11-04

**Authors:** Glenn Jenkins, Kenneth J. O'Byrne, Benedict Panizza, Derek J. Richard

**Affiliations:** ^1^University of Queensland, Brisbane, QLD, Australia; ^2^Cancer and Ageing Research Program, Institute of Health and Biomedical Innovation at the Translational Research Institute, Queensland University of Technology, Brisbane, QLD, Australia; ^3^Princess Alexandra Hospital, Brisbane, QLD, Australia

## Abstract

Genomic instability underlies the transformation of host cells toward malignancy, promotes development of invasion and metastasis and shapes the response of established cancer to treatment. In this review, we discuss recent advances in our understanding of genomic stability in squamous cell carcinoma of the head and neck (HNSCC), with an emphasis on DNA repair pathways. HNSCC is characterized by distinct profiles in genome stability between similarly staged cancers that are reflected in risk, treatment response and outcomes. Defective DNA repair generates chromosomal derangement that can cause subsequent alterations in gene expression, and is a hallmark of progression toward carcinoma. Variable functionality of an increasing spectrum of repair gene polymorphisms is associated with increased cancer risk, while aetiological factors such as human papillomavirus, tobacco and alcohol induce significantly different behaviour in induced malignancy, underpinned by differences in genomic stability. Targeted inhibition of signalling receptors has proven to be a clinically-validated therapy, and protein expression of other DNA repair and signalling molecules associated with cancer behaviour could potentially provide a more refined clinical model for prognosis and treatment prediction. Development and expansion of current genomic stability models is furthering our understanding of HNSCC pathophysiology and uncovering new, promising treatment strategies.

## 1. Introduction

**Figure 1:**
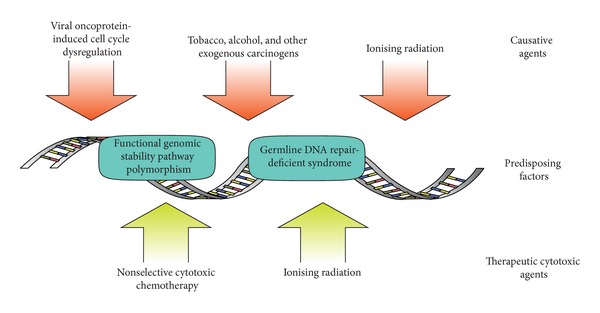
Common genomic stressors in carcinogenesis and therapy.

Carcinogenesis and evolution of the cancer genome are driven by genomic instability. We review here advances in our understanding of the pathways that preserve genome integrity that have improved insight into cancer behaviour, prediction, prognosis, and personalised therapy. Traditional anticancer therapy has exploited the inherent genomic instability of malignancy; however, this mutagenic pressure also promotes the emergence of treatment resistance, invasion, and metastasis (Figure 1). Squamous cell carcinoma of the head and neck (HNSCC) is the sixth most-common cancer in the developed world [1, 2] and represents a therapeutically-challenging, behaviourally-heterogenous category of disease. Genomic instability is a defining characteristic of HNSCC [3]. Subregional differences in patterns in risk, treatment response, and prognosis in HNSCC are underpinned by aetiological factors that affect genomic stability in different ways. In HNSCC, the principal subsites of the upper aerodigestive tract are oral cavity (including tongue, floor of mouth, and buccal surface), nasopharynx, oropharynx (including tonsil and base of tongue), and larynx. An important emergent epidemiological change in HNSCC has been the increasing prevalent of human papillomavirus- (HPV-) associated cancer. 

Chemotherapy regimens in HNSCC are based on platinum compounds, prototypically cisplatin, with or without 5-fluorouracil [4], with greatest effect when given concurrently with radiotherapy [5, 6]. The absolute 5-year survival benefit conferred by concurrent cisplatin/5-fluorouracil chemotherapy is a modest 4.5% [5, 7] and is associated with significantly increased treatment morbidity and mortality [8]. Newer chemotherapeutic agents include taxols and epidermal growth factor receptor- (EGFR-) inhibitors [9]. Addition of docetaxel, a mitotic spindle stabilizer, to PF induction chemotherapy has been shown to confer an additional survival benefit [10], although heterogeneity between trial treatment arms and high loss-to-followup make absolute benefit difficult to interpret. EGFR inhibitor therapy has emerged as an effective adjuvant in HNSCC and is discussed in detail below.

Chromosomal mutation is one of the most readily observable features of cancer and has been known to underpin malignancy for over a hundred years [11]. The mechanism behind most common chromosomal lesions is genomic instability, which is influenced by the interdependent triad of accumulating DNA damage, defective DNA damage repair, and replication stress. In HNSCC, DNA damage is increased by exposure to carcinogens (tobacco, alcohol, and various regionally specific botanicals). Ineffective repair by a number of germline variations in the DNA repair pathways, long recognised in congenital repair syndromes such as Fanconi anemia, have been increasingly implicated as factors in HNSCC, particularly in phenotypically silent individuals with single-nucleotide polymorphisms (SNPs) of DNA repair genes. The DNA damage response is a highly conserved pathway, that is, activated on the basis of a “threshold” of DNA damage events and functions to inhibit cell cycle progression via checkpoint signalling for either repair or apoptosis as well as directly in the repair of the lesions. These pathways are known to be constitutively active in dysplastic lesions [12–14]. This genetically unstable environment drives mutagenic stress, with selective pressure for inactivation of growth restricting/apoptotic processes [13, 15]. Replication stress drives the accumulation of DNA lesions in HNSCC, and is promoted by genetic loss of cell-cycle checkpoint control through mutation and epigenetic loss via oncoviral coinfection with high-risk human papillomavirus (HPV). 

Head and neck squamous-cell carcinoma has a male predominance and is associated with tobacco, alcohol, and use of a number of alkaloid-rich regional stimulants (such as betel nut). A subset of HNSCC is strongly associated with “high-risk” HPV subtypes 16 and 18 infection [16] that are present in up to 60–80% of nonoral HNSCC and 30% of oral cavity SCC in a Western population [17–20]. This group of disease that affects younger patients is proportionally associated with number of sexual partners and has a distinctly improved prognosis and different treatment profile from HPV-unrelated oropharyngeal HNSCC [21–27] as well as a distinct pattern of chromosomal mutation [28, 29]. Prognostic significance in oral cavity SCC is less clear (reviewed in [19]). The association of HPV with oral squamous-cell carcinoma was observed 30 years ago [30]; however, the epidemiological changes became more evident over the following decades as decreased tobacco use and increased HPV prevalence highlighted this subgroup [16]. Viral oncoproteins E6 and E7 have been implicated as the causative agents in HPV-associated HNSCC. E6-induced ubiquitin-mediated proteosomal degradation of tumour suppressor p53 causes cell-cycle dysregulation and increased replication stress [31–33]. Oncoprotein E7 binds to and disrupts pRb-mediated degradation of transcription activator E2F, which promotes genes responsible for cell-cycle progression [34–36]. Tumour suppressor protein p16, a CDK4A inhibitor, is characteristically elevated in E7-induced pRb suppression, to the extent that p16 levels are typically used as a surrogate marker for HPV-related SCC [37–39]. E7 has also been shown to induce aberrant centrosome number and mitotic spindle formation [40, 41]. These proteins function to allow viral replication in the normally postmitotic upper differentiated epithelial layers [42]. Whole-exome sequencing has shown that HPV-related HNSCC is associated with less instability, with a 2-fold lower mutation rate [43]. Interestingly, while suppression of the upstream DNA damage response ataxia-telangectasia mutated protein (ATM) and ataxia-telangectasia and Rad3-related protein (ATR) is deleterious for HPV episome stability [44]; cell line studies have shown that the radiosensitivity of HPV-16 related SCC is attributable in part to a repair defect with accumulation of unrepaired DNA double strand breaks and resultant cell-cycle arrest [45]. This study was conducted using a limited number of cell lines rather than fresh tissue; however, the relative contribution of HR and NHEJ in HPV-related SCC has yet to be explored. 

While p16 is characteristically elevated in HPV-associated oropharyngeal cancer, up to 56–63% of OSCC and 59% of premalignant leukoplakia lesions show specific downregulation of p16, as expected of the tumour-suppressor role it has [46–49]. HPV-associated oral cavity SCC shows a lower incidence of p16 positivity (65% high risk HPV c.f. 44% low-risk/HPV negative cancers) [50], as does hypopharyngeal cancer (11% high-risk HPV c.f. 0% low-risk/HPV negative cancers) [51]. CDK2NA (encoding p16) is commonly inhibited via promoter hypermethylation, and this is associated with a worse prognosis [52, 53]. 

## 2. Chromosomal Mutations in HNSCC

One of the most morphologically apparent features of neoplasia is chromosomal mutations and rearrangements, and these have been well described in HNSCC. Flow cytometric analysis of both dysplastic and malignant lesions shows the expected derangement of chromosomal ploidy. Clinically precancerous lesions show rates of 46% diploidy, 37% tetraploidy, and 17% aneuploidy, while malignant lesions show 10% diploidy and 90% aneuploidy [54]. In addition to deletions and duplications (copy number variation, CNV), a critical component of carcinogenesis is allelic loss or loss-of-heterozygosity (LOH). 

The first chromosomal aberrations in premalignant lesions are losses of 3p, 9p, 5p, and 17p [55–57]. Importantly, CDKN2A encoding p16 is localised to the most commonly affected 9p21-22 locus [58, 59], which shows LOH in 46–71% of premalignant lesions and 72% of carcinoma [59, 60] and is strongly associated with progression to cancer and metastasis [60, 61]. Lydiatt et al. have suggested that this deletion is overrepresented in HNSCC cell lines [62], although this study found much lower rates of primary tumour deletions. While germline p16 mutations have been described in familial HNSCC syndromes, these are rare [63, 64]. 

Allelic loss of 3p is present in 74–81% HNSCC [56, 65, 66], with PCR and hybrid clone studies suggesting three discrete areas at 3p13-p14 3p21.2-p21.3, and 3p25 [65, 67] are positively associated with tobacco-related disease and nodal status [68]. The FHIT gene encoding a common epithelial tumour suppressor is found at locus 3p14.2. Expression of FHIT is suppressed in 65% of HNSCC and is associated with worse overall survival [69]. Locus 3p21.31 contains a number of tumour-suppressor genes (*LIMD1, LTF, CDC25A, SCOTIN, RASSF1a, *and* CACNA2D2*) of which alterations to LTF and RASSF1A were associated with significantly worse outcome in oral cavity disease in an Indian cohort [70]. 3p25 contains the gene locus for hOGG1 and von Hippel-Lindau tumour suppressor gene. hOGG1 is an important component of the base excision DNA repair pathway and shows LOH in 55–61% of HNSCC and is underexpressed in 49% [71, 72]. As discussed below, this mechanism is important in the repair of ionizing radiation and common oxidative and tobacco-related DNA lesions [73]. Unrepaired tobacco-induced benzo[*a*]pyrene lesions commonly result in G:T transversion mutations [74, 75]. Repair of the oxidative lesion 8-oxoG also relies on this pathway, which can result in a stable G:C to 8oxoG:A missense substitution when encountered by the DNA polymerase [76]. Together these substitutions comprise 54% of the most common inactivating p53 point mutations in HNSCC [77]. This mutation pattern, which has also been strongly linked to smoking-induced lung cancer [78], is correlated with smoking exposure-related HNSCC [43].

Chromosomal aberrations associated with late disease and metastasis are less well characterised. It has been found that metastatic deposits mostly retain clonality with their primary cancers [79]. Gains of 3q, 11q13, 7q11.2, and 1q21-q22 and losses of 8p, 11p14, 10p12, 10q, and 14q were reported in association with metastasis by Bockmühl et al. [79, 80], who also found a high rate of 45% deletions in the 8p23 region correlated with poor prognosis [81]. This region corresponds to putative tumour suppressor gene CUB and SUSHU multiple domain protein 1 (CSMD1) which is associated with a high risk, although higher-resolution deletion mapping has shown inconsistent targeting of this gene [82]. Many more chromosomal mutations have been described in later diseases [61, 83–89]. These rearrangements occur at common fragile sites, late-replicating regions which can preferentially stall replication forks under conditions of replication stress [90]. Inappropriate recovery of these forks can cause double-strand DNA breaks or inversions [91], and ineffective repair can lead to translocations and deletions [92, 93]. For more detail on other chromosomal rearrangements see [94, 95] for a comprehensive review. 

## 3. The DNA Damage Response

**Figure 2:**
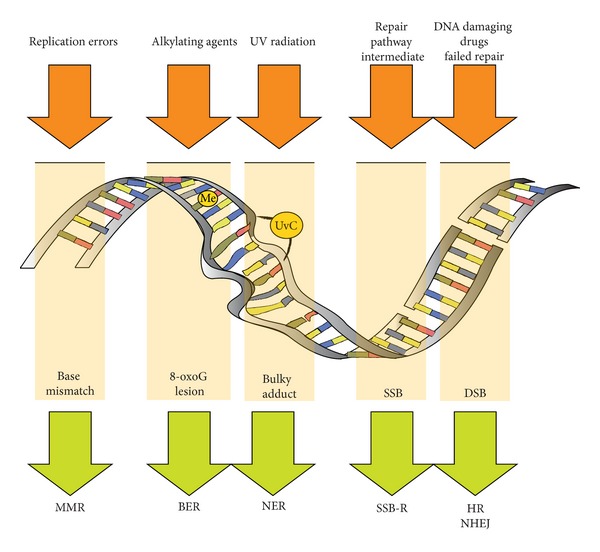
Examples of DNA lesions and repair pathway choice.

Highly conserved across all domains of life is a robust DNA damage recognition and repair system. Genomic material, both DNA and RNA, is under constant degradation from intrinsic and extrinsic factors. These include oxidative stress, ultraviolet radiation, ionising radiation, and other chemical alterations such as alkylation [96, 97]. Cells under active division, such as deregulated tumour cells, are particularly sensitive. This differential sensitivity is exploited by chemotherapy and external-beam radiation. There are many different types of DNA lesions, which are broadly dealt with via interacting pathways (Figure 2):

1. mismatch repair (MMR),
2. nucleotide excision repair (NER),
3. base excision repair (BER),
4. single-strand break repair (SSB-R)*,
5. double-strand break repair (DSB-R).

Single-strand break repair is not usually considered a distinct repair pathway and is integrated into BER, NER, and DSB-R. The most severe form of DNA damage is the double-strand break [98]. Base excision repair and nucleotide excision repair pathways involve single-strand break intermediates, which unrepaired become double-strand breaks when encountered by a replication fork [12]. Double-strand break repair occurs via two pathways nonhomologous end-joining (NHEJ) and homologous recombination (HR). The primary pathway in nonreplicating mammalian cells is NHEJ [12].

ATM is an upstream phosphatidylinositol 3 kinase-like kinase (PIKK), that is, recruited to double-strand break sites and coordinates repair. Limited studies in ATM have shown a strong increased cancer risk with a specific SNP in an East Asian population [99]. Promoter hypermethylation of ATM has been linked with early age of onset and poor prognosis in one mixed subsite study [100]; however, this has not been reproduced in a large oral cavity cancer series [101]. The mutation most common in the ataxia-telangectasia phenotype does not seem to be a significant contributor to the development of HNSCC [102].

## 4. Mismatch Repair Pathway

Microsatellite instability is a distinct, measurable form of genomic damage, that is, linked to the mismatch repair pathway, principally involving mut-S homologin 2 (MSH2) and mut-L Homologin 1 (MLH1). The archetypical germline defect in this pathway is the hereditary nonpolyposis colorectal carcinoma syndrome [103]; however, the involvement of MSI in HNSCC has been less clearly elucidated. Early studies showed that direct mutation or deletion of hMLH1/hMSH2 was uncommon [104, 105], and reported frequency of MSI in HNSCC has ranged from 7% to 100%, varying with marker choice, tumour site, and patient demographic [104–109]. MSI has been found to be more prevalent in younger patients [104], and Ha et al. have shown that it becomes more frequent as dysplastic lesions progress to malignancy (14% and 55%, resp.) [110].

The dominant moderator of mismatch repair in HNSCC is promoter hypermethylation rather than direct mutation [111]. MLH1 promoter hypermethylation has been found in 76% of one OSCC series, associated with early disease, where 38% of tumours exhibited protein underexpression [112]. There is however wide variation in the reported prevalence of promoter methylation, from 8% to 69% [113–115], and normal tissue demonstrates promoter methylation of up to 45% [115]. Most studies show no correlation with tobacco exposure; however, there may be an association with metachronous primary cancers [114]. A more recent study of MLH1, MSH2, and MGMT in mobile tongue SCC showed increased expression of the mismatch repair proteins (55.1% MSH2, 36.73% MLH1), which was associated paradoxically with less muscle invasion, less perineural invasion but lower overall survival, higher rates of lymphatic metastasis, and more aggressive morphology, implying that this may be a reactive rather than causative finding [116]. 

## 5. Base Excision Repair Pathway

hOGG1 and NEIL1 and 2 are DNA glycosylases that are involved in the first step of damage recognition. They demonstrate significant functional cross-over with each other [117] and show affinity for lesions from 5-fluorouracil (5-FU), UV damage, and 8-oxoguanine (a highly mutagenic by-product of ROS). The hOGG1 locus commonly undergoes LOH as discussed above, with up to 49% of HNSCC demonstrating underexpression [72, 118]. Interestingly, there is some evidence that this defect is present systemically and may be due to a germline rather than sporadic mutation [119], although haplotype studies have been inconclusive as shown above. APEX1, a processing enzyme immediately downstream of hOGG1, has been shown to be overexpressed in HNSCC in a Pakistani population and is associated with nodal positivity and later stage [120]. NEIL1/2 form a redundant BER system, substituting AP endonuclease (APEX1/2) for polynucleotide kinase (PNK) [121], and the g.4102971CC polymorphism has been linked to increased risk of HNSCC [122]. 

XRCC1, as discussed above under SNPs, has been extensively investigated for its role in HSNCC. It functions in DNA single-strand break repair downstream of APEX1/PNK to scaffold and coordinate nucleotide synthesis [123]. Early studies of mRNA expression in head and neck cell lines implied that XRCC1 was not a significant contributor to cancer radiosensitivity [124]; however, a more recent protein expression series in primary laryngeal cancer looking at outcomes following definitive radiotherapy has contradicted this [125]. Overexpression of XRCC1, in a more recent series, has been linked to poor outcome in chemoradiation-treated HNSCC but not other modalities [126], and other studies have reported a predominant pattern of underexpression in an Indian population [118].

Poly- (ADP-Ribose) polymerase (PARP) 1 and 2 are DNA repair proteins that have direct affinity to many DNA lesions and once bound autocatalyze the generation of a PAR polymer which induces chromatin decondensation and recruits many other repair molecules, including ATM [127]. Although it has no direct repair role, it is required for efficient base excision repair, nucleotide excision repair, HR, and alternative-NHEJ [127–131], with the most well-understood role of single-strand break binding to facilitate polymerisation and ligation. Of particular importance in malignancy, PARP-1 is required for safe traversal of damaged DNA by the replication machinery and subsequent HR-mediated repair [128], which is significantly exacerbated under replication stress. Importantly, PARP inhibition has been found to be synthetically lethal in BRCA- (HR-) deficient breast cancer [132, 133]. As discussed above, EGFR-inhibition induces an HR and NHEJ deficiency in addition to antiproliferative effects. There is evidence that combined therapy can replicate an HR “block” in a similar fashion [134] that may be linked to Nbs1 suppression [135, 136]. Indeed, cosuppression of many proteins involved in HR induces *in vitro *PARP susceptibility [137]. Blocking upstream signalling by PI3K also induces HR defects and PARP-sensitive synthetic lethality in breast cancer cell lines [138]. There is also limited data in HNSCC demonstrating a link between PARP1 overexpression and cisplatin resistance [139], suggesting a possible role for chemoresistant tumours. 

## 6. Nucleotide Excision Repair Pathway

The nucleotide excision pathway (NER) corrects “bulky” DNA adducts and lesions such as UV photoproducts, platinum lesions, and tobacco-linked lesions [140]. Germline defects in this pathway are responsible for the xeroderma pigmentosum phenotype, for which many key proteins are eponymous. There are two principal monitoring pathways, global genomic NER and transcription-coupled NER.

Global genomic NER is initiated by direct recognition of DNA lesions irrespective of transcription [141, 142], and polymorphism of several key proteins have been implicated in HNSCC as shown above. Transcription-coupled NER differs in that detection and recruitment of repair molecules is initiated by stalled RNA polymerase II (RNAP II), with repair mediated by UVSSA-USP7 and BRCA1 [143, 144]. The lack of a clear association with a cancer syndrome, *in vitro* or *de novo* cancers make the transcription-coupled pathway a less promising factor in cancer genome stability [145, 146]. 

ERCC1-XPF has been extensively studied in lung cancer, breast cancer, and HNSCC. High ERCC1 expression in HNSCC has been associated with cisplatin resistance and poor survival in a betel chewing endemic area [147]. Two larger retrospective trials showed prognostic benefit of low ERCC1 expression in HNSCC (HR 0.42, CI_95%_ 0.20–0.90, *P* 0.03 and HR 0.12, *P* 0.043) [148, 149]. *In vivo* ERCC1 has been associated with enhanced cisplatin resistance [150] and is involved in alternative NHEJ [151]. Metastasis has also been associated with increased expression of XPF [152].

SNPs in all eight major NER genes have been implicated in increasing background risk of oral leukoplakia [153], and altered PBL expression of ERCC1, ERCC5, ERCC6/CSB [154], and ERCC4 [155] have been found to be higher in patients with oral cancer. Similarly to peripheral blood lymphocyte assays done in hOGG1 (discussed above), there is limited evidence that there may be a systemic NER deficient phenotype in a small cohort of patients with HNSCC [156]. Some SNPs have been implicated as protective or hazardous in single SNP studies (refer Table 1), while multiple (5–7) risk NER genotypes have been associated with a 2.4-fold increased relative risk of second primary HNSCC [157].

## 7. Nonhomologous End Joining Pathway

The Ku heterodimer is comprised of Ku70 and Ku80 functions as a sensor, binding to free dsDNA ends, an end-processing 5′-dRP/AP lyase, and a recruiter of DNA-PKcs [158] and telomere protection [159]. Ku80 overexpression has been linked to poor prognosis, local failure, and recurrence following radiation [160]. While this study did not include tongue cancer specimens, the predictive value was greatest in HPV negative cancers (RR 9, *P* < 0.01). In contrast, two similarly-sized mixed studies found Ku70 mRNA levels and protein overexpression improved recurrence-free survival in locally advanced HNSCC [161] and tonsillar cancer [162]; however, these did not stratify for p16 status and had unusual treatment regimens. In support of these findings are *in vitro* studies showing that Ku70 loss improved radioresistance in late G2/S, possibly by allowing the more error-free HR [163]. 

## 8. Homologous Recombination Pathway

The Fanconi anemia pathway encompasses eleven proteins involved in the HR-mediated repair of interstrand crosslink (ICL) lesions [164]. Fanconi anemia homozygous patients have a much greater rate of head & neck solid tumours [165], and underexpression of many Fanconi proteins is common in primary HNSCC [166], particularly in young patients [167], although the underlying mechanism is unclear. Tobacco exposure has been shown to selectively suppress FANCD2 expression in a respiratory epithelial cell line [168]. Promoter hypermethylation of FANCF has been found in 15% of HNSCC [169], while 42% of OSCC show FANCC methylation in both malignant and premalignant lesions in an Indian population [170]. In contrast, another series demonstrated 31% PHM of FANCB, and found other Fanconi genes to be rarely methylated [171]. While cisplatin commonly induces interstrand crosslinks, specific upregulation of the Fanconi pathway has not been implicated in cisplatin-resistant HNSCC cell lines [172].

Hyperphosphorylation of replication protein A (RPA), a single-strand DNA binding protein, that is, integral to HR, has been implicated as a mechanism for cisplatin resistance in HNSCC cell lines [173]. RPA signalling activity is heavily modulated by phosphorylation status, and this posttranslational resistance mechanism promotes inappropriate cell-cycle progression.

BRCA1 and 2 are essential for efficient HR, and germline mutation is causally linked to breast and ovarian cancers [174, 175]. Low BRCA expression has been correlated with better cisplatin response and survival in lung cancer [176]. BRCA-deficient breast cancers have shown survival benefits with synthetically lethal PARP inhibition in Phase II trials [177]. An interesting pattern in tongue SCC pathogenesis shows BRCA1 overexpression in leukoplakia followed by subsequent underexpression in carcinoma [178]. Sporadic mutation of BRCA2 is rare [179] as is promoter hypermethylation [169]. 

## 9. The Epidermal Growth Factor Receptor in DNA Repair

**Figure 3:**
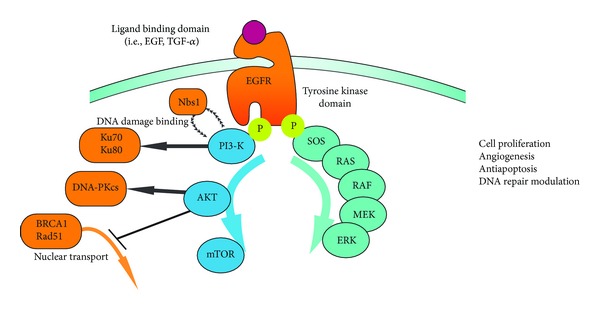
Downstream effects of EGFR on DNA repair proteins.

Epidermal growth factor receptor (EGFR) is a transmembrane signalling protein belonging to the receptor tyrosine kinase family, which has significant implications in HNSCC. EGFR is physiologically activated via ligand binding (transforming growth factor-*α*, TGF-*α*), and active EGFR promotes cell proliferation and enhances radioprotective mechanisms through the mitogen-activated protein kinase (MAPK) pathway and the extracellular signal-regulated kinases (ERK1/2). [180, 181]. The strongest effect of EGFR activation on DNA repair is via upregulation of the phosphoinositol-3 kinase (PI3K)/AKT signalling pathway (Figure 3). PI3K mutations are common in HNSCC and linked with higher genomic instability [182]. PI3K promotes transcription of the nonhomologous end-joining complex DNA-PK [183, 184], mediates Nbs1 binding for DNA damage detection [185], and promotes effective DNA double strand break repair [186], while AKT1 promotes transcription of the DNA-PKcs subunit [187] and negatively regulates BRCA1/Rad51 nuclear transport [188]. Particularly relevant to DNA repair, inhibition of the EGFR receptor induces a NHEJ and HR defect. Erlotinib has been shown to decrease BRCA1-dependent HR by twofold in breast cancer cell lines [189] and attenuate radiation-induced Rad51 expression (a key HR protein) [190]. Gefitinib delays Nbs1 recruitment to DNA double strand break sites in lung cancer cell lines and significantly reduces DNA double strand break repair [136]. Downstream inhibition of PI3K has also been shown to induce a BRCA1/2 deficiency in breast cancer cell lines [138]. The functionality of the BRCA/HR pathway is of particular importance for the utility of PARP inhibitors (discussed below).

Constitutively-active inhibitor-sensitive EGFR mutants are an important mechanism in 10% of nonsmall cell lung cancer (NSCLC) cases, conferring a significant survival benefit when cancer cells dependent on this proliferative signal are deprived via EGFR-inhibition [191, 192]. Such kinase domain mutations are rare however in HNSCC, with mutation rates of 7–16% in Asian and 0–4% in Japanese and Caucasian populations and are not present in HPV-positive tumours [193–197]. This suggests that they do not have a significant role in oncogenesis and may instead reflect underlying instability. In striking contrast, an EGFR inhibitor-resistant truncation mutant EGFRvIII has been found in up to 42% of HNSCC [198]. This mutant has a deletion of exons 2 to 7, involving the extracellular binding domain rather than the kinase domain [199]. This results in a protein, that is, constitutively active, has altered kinase dynamics that favour PI3K signalling, and that promotes ligand-independent invasion and migration [200, 201]. 

Oncogenic mutations in other components of this pathway are less common. PI3KCA somatic mutations have been described in 0–11% of HNSCC tumour samples and are overrepresented in cell lines [202–204], while PTEN and Akt1 mutations are rare [205]. 

Overexpression of EGFR is very common in HNSCC, with protein immunohistochemical studies showing high expression in 43–68% of HNSCC [197, 206–208] and Areca quid-associated OSCC demonstrating a lower rate of 23% [209]. High EGFR protein levels are strongly linked to poor cause-specific survival [207, 208], nodal stage and dedifferentiation [197], and emergence of stem-cell like characteristics [210]. Altered EGFR copy numbers have been found in 24% of HNSCC cases in one study (17% increased, 7% decreased) and both are linked to a reduced cause-specific survival and disease-free survival [211]. EGFR amplifications have been demonstrated in 24% (10% deletions) of laryngeal cancers, although survival significance was only demonstrated when combined with chromosome 7 copy-number status [212]. There does not appear to be any difference between HPV-associated and HPV-negative cancers with regard to EGFR expression [50]. 

Cetuximab, an IgG1 monoclonal antibody against the receptor domain of EGFR, has been shown to add an 8% absolute 2-year survival advantage as a monotherapy adjunct to radiotherapy in HNSCC [213] and has become incorporated into the standard management of late-stage HNSCC [214]. The tyrosine kinase inhibitor gefitinib has also been shown to sensitize HNSCC cells to cisplatin via destabilization of Rad51 [215]; however, Phase III trials have shown no advantage as monotherapy in recurrent HNSCC compared with methotrexate [216]. Cancers with high EGFR expression demonstrate additional benefit from hyperfractionated radiotherapy regimens [217]. 

Radioprotection in EGFR-competent cells is improved via enhanced immediate DNA repair, protection from damage-induced apoptosis, and enhanced proliferation of the EGFR-competent subpopulation during repopulation [218], providing a selective advantage. Cetuximab resistance has been linked to EGFR translocation from the cytoplasm to the nucleus and endogenous ligand production [219]. Transport dysregulation and HER2-dependent transactivation has also been found to contribute to resistance in lung cancer cell lines [220]. Emergent resistance is also very common in colorectal cancer, where it has been found to be mediated by endogenous upregulation of K-Ras [221].

## 10. Single Nucleotide Polymorphisms in DNA Repair Pathways

While some areas of genomic DNA are highly conserved, the dynamic variability of large domains encompassing coding and noncoding areas is becoming increasingly important to our understanding of individual disease risks. The most common examples are single nucleotide polymorphisms (SNPs), of which 3.1 million have been identified encompassing most proteins involved in DNA repair [222]. The two principal types of SNPs are synchronous (or “silent”) and nonsynchronous, where a base substitution results in a corresponding protein substitution. The protein product may be a “functional variant,” or has some degree of impaired function. Analogous to hereditary DNA repair syndromes, much attention has been paid to SNP prevalence and the effect on HNSCC risk. Listed below are a subset of SNP studies specific for DNA repair proteins involved in the pathways discussed above.

In addition to the above studies, a series in a Pakistani population found a remarkably high prevalence of novel hOGG1 polymorphism is association with HNSCC, although this has not been subsequently validated [223]. Interestingly, both this and a Japanese study found a statistically significant link between hOGG1 Ser326Cys status and tobacco use [224], complicating statistical analysis. Likewise, HPV-16 positivity may alter the significance of XRCC1 polymorphism in HNSCC risk, possibly explaining the inconsistent results between study populations [225, 226], although these findings are in contrast to cervical SCC and HPV-16 status where no relationship has been found [227]. Complex gene-gene interactions between XRCC1 and XPD haplotypes have also been described [228].

Carles et al. [229] examined nine nucleotide excision repair gene SNPs with regard to response to definitive radiotherapy. This study found XPA (5′UTR), XPF/ERCC1 C259C, and XPG/ERCC5 G1104C/T46C SNPs resulted in a significantly worse overall survival following treatment in a cohort of 104 patients over 10 years. In a Brazilian population, ERCC1 T19007C was not found to affect response to treatment or overall survival in a modest cohort [230]. 

## 11. Discussion

Genomic instability underpins the development of dysplasia, malignancy, invasion, and metastasis in cancers. Many of our cancer therapeutic drugs exploit these genetic stability pathways by adding extra pressure onto already primed cancerous cells. The formation of these cancers is also clearly linked to genetic instability with the high risk factors such as HPV, tobacco, areca nut, betel quid, and alcohol having direct mutagenic effects on the cellular genome. There is also an emerging spectrum of DNA deficient high risk phenotypes that are improving our understanding of background genetic risk. These aetiological differences in oncogenesis translate into distinctly different disease profiles. Profiling of the DNA repair pathways in established cancer may allow the development of robust biomarker-based cancer phenotyping for both improved prognosis and personalized therapeutic selection. The success of the PARP-1 inhibitor olaparib in BRCA1-deficient breast cancer and the early translational work in HNSCC underpin the importance of future targeted therapy in exploiting synthetic lethality in DNA repair. 

The inherited DNA repair deficient syndromes provide the prototype for insight into DNA repair in cancer, highlighting the importance of the double-strand repair pathways, and in particular homologous recombination. Ataxia telangiectasia presents with a significant increased all-cause cancer risk [231] consistent with its critical repair role and inherited HR protein BRCA1 and BRCA2 mutants strongly predispose toward breast cancer, prostate, and colon cancer [175]. Homologous recombination is the preferred repair pathway in cells undergoing active replication and is constitutively active in cancer. Defective HR is responsible for many chromosomal rearrangements [91, 232], and emerging evidence shows a robust redundant pathway, that is, more complex than previously thought. Complementing our knowledge of this pathway is ongoing discovery of HR-essential proteins such as hSSB1/2 [233, 234], RNF8 [235], and Exo1 [236], which coordinate the more well-understood core repair complexes such as Rad51 and MRN [237]. Further exploration of the oncogenic role of these core proteins is needed.

Head and neck cancer is a diverse category of disease, with outcomes influenced largely by primary site and p16 status. Existing studies on more well-known DNA repair biomarkers are often difficult to interpret due to conflation of anatomical subsites or limited p16 control. Focused, well-powered studies controlled for anatomical subsite are likely to be of greater clinical benefit. Similarly, the abundance of the literature on genetic polymorphism and cancer risk is tempered by conflicting reproducibility, conflation of anatomical sites, and population differences. Localisation of SNP studies is important given the significantly unique risk factor profiles in East Asian and Chinese populations; however, it makes generalisation of these highly prognostic studies to Western populations difficult.

Current gaps in the literature of cancer genomic instability include the nature of interpathway and intrapathway redundancy. Major protein complexes can be less efficiently replaced by alternate, multipurpose proteins to otherwise allow DNA repair [235, 238], an important mechanism to understand in designing synthetically lethal repair blockade. SNPs of backup 8oxoG pathways such as PNK/NEIL1 have been linked to HNSCC risk [122] and contributes to poor survival in non-HNSCC cancer [239]; however, the role of this pathway in relation to hOGG1 deficiencies has yet to be determined in HNSCC.

Improved understanding of the nature of genomic instability in head and neck cancer has helped clarify the interplay between mutagenic stresses and DNA repair efficiency. Future characterization of signature instability patterns will guide further therapeutic development targeted at the critical foundation underlying malignancy.

## Figures and Tables

**Table 1:** 

Reference	Subsite	Population	Study size	Gene SNP	Odds ratio (*P* value)
Sliwinski et al. [240]	Laryngeal	Polish	641 (288 cases, 353 controls)	Rad51 G135C XRCC3 C722T	2.92 (<0.0001) 4.03 (<0.0001)

Romanowicz-Makowska et al. [241]	Laryngeal	Polish	506 (253 cases, 253 controls)	Rad51 G135C XRCC2 G31479A XRCC2 C41657T	NS [*3.43 (0.073)*] NS [*3.81 (0.061)*] NS [*2.14 (0.061)*]

Werbrouck et al. [242]	All	Belgium	169 cases, 352 controls	XRCC3 C722T Rad51 G-3429C Lig4 C26T Ku70 C1310G Ku80 c.2110-2408 G>A	1.96 (0.02) 0.43 (0.05) 0.43 (0.01) NS NS

Cui et al. [243]		North American	443 cases (including oesophageal), 912 controls	XPG c.1104	0.47 (<0.05)

dos Reis et al. [244]	Oral cavity	Brazil	300 (150 cases, 150 controls)	XRCC1 Arg194Trp, Arg399Gln XRCC3 Thr241Met	NS NS

Pawlowska et al. [245]	Laryngeal	Polish	506 (256 cases, 256 controls)	hOGG1 Ser326Cys	2.96 (0.012)

Kietthubthew et al. [246]	Oral cavity	Thai (betel quid endemic)	106 cases, 164 controls	XRCC1 Arg194Trp, exon 6	NS
				XRCC3 Thr241Met	3.3 (0.01)

				XRCC3 rs3212057	6.0 (<0.01)
Gresner et al. [247]	All	Polish	81 cases, 111 controls	Rad51 rs5030789 AA	0.33 (<0.05)
				Rad51 rs1801321 TT	0.1 (<0.05)

Zhang et al. [248]	All	North American	706 cases, 1196 controls	hOGG1 Ser326Cys	NS

Elahi et al. [249]	All	North American	169 cases, 338 controls	hOGG1 Ser326Cys Cys326Cys	1.6 (<0.05) 4.1 (<0.05)

Majumder et al. [225]	All	Indian	180 cases, 150 controls	XRCC1 c.194, c.399	NS

Mahjabeen et al. [120]	Not specified	Pakistani	300 cases, 300 controls	APEX1 13T<GG Ser129Arg Val131Gly	4.97* (0.0001) 11.74* (0.0001) 5.12* (0.0001)

Ramachandran et al. [250]	Oral cavity	Indian	110 cases, 110 controls	XRCC1 c.194 c.280 c.399 XPD c.751	3.09 (<0.0001) NS 2.37 (<0.0001) 2.10 (0.003)

Tae et al. [251]	All	Korean	147 cases, 168 controls	XRCC1 R194W R280H, R399G	2.61 (<0.05) NS

Kumar et al. [118]	Not specified	North Indian	75 cases, 75 controls	XRCC1 Arg399Gln XPD Lys 751Gln hOGG1 Ser326Cys	NS

Kumar et al. [228]	Not specified	North Indian	278 cases, 278 controls	XRCC1 Arg194Trp Arg280His Arg399Gln XPD Lys751Gln	0.72 (0.03) NS 0.64 (0.003) 1.75 (0.002)

Kowalski et al. [252]	All	Polish	92 cases, 124 controls	XRCC1 Arg194Trp Arg399Gln	NS

Yen et al. [253]	Oral cavity	Taiwan	103 cases, 98 controls	XRCC1 Arg194Trp XRCC2 (5′ locus) XRCC3 Thr241Met XRCC4 T139G	NS
				“pseudo-haplotype” multiple concurrent SNPs	2–2.45 (0.03) 3–5.03 (0.013) 4–10.1 (0.036)

Tseng et al. [254]	Oral cavity	Taiwan	318 cases, 318 controls^(1)^	XRCC4 c247 G1394T rs28360317 rs1805377	2.04 (<0.05) NS NS NS

Chiu et al. [255]	Oral cavity	Taiwan	318 cases, 318 controls^(1)^	XRCC4 G1394T rs28360071 rs28360217 rs1805377	NS 1.55 (<0.05) NS NS

Chiu et al. [256]	Oral cavity	Taiwan	292 cases, 290 controls	ERCC6 c.399 (A/A) c.399 (G/G, G/A) c.399 (Any A) c.1097 c.1413	1.82 (<0.05) NS 1.43 (<0.05) NS NS

Hsu et al. [257]	Oral cavity	Taiwan	600 cases, 600 controls^(1)^	Ku80 G-1401T C-319T Intron19	1.603 (<0.05)^(2)^ NS NS

Bau et al. [258]	Oral cavity	Taiwan	318 cases, 318 controls^(1)^	Ku70 T991C G-57C A-31G Intron 3	2.15 (<0.05) NS NS NS

Bau et al. [99]	Oral cavity	Taiwan	620 cases, 620 controls	ATM rs189037 rs600931 rs652311 rs228589 rs227092 rs227060	1.61 (<0.05) NS NS NS NS NS

Flores-Obando et al. [259]	Meta-analysis	Multiple		XPA A23G XPD C22541A/A XPD A35931C XPD Asp312Asn XPD C23047G XPD C23051G XPC PAT XPC Lys939Gln XPC Ala499Val XPF T2063A ERCC1 C8092A XRCC1 exon 6 T/T XRCC1 exon 10 XRCC1 exon 9 XRCC3 Thr241Met	NS 0.74 (<0.05) NS 1.14 (0.05) NS NS NS NS 1.56 (<0.05) NS NS 1.69 (<0.05) NS NS NS

Zhai et al. [122]	All	North America	872 cases, 1044 controls	NEIL1 g.46434077 NEIL1 g.46438282 NEIL2 g.4102971CC	NS NS 1.30 (<0.05)

*Crude odds ratio.

(1) Same group/cohort.

(2)Only in Areca-nut exposed subgroup.
